# Determinants of continuous visiting behavior to Palawan, Philippines: Integrating Uncertainty Reduction Theory and Expectation Confirmation Theory

**DOI:** 10.1371/journal.pone.0291694

**Published:** 2023-10-03

**Authors:** Maela Madel L. Cahigas, Yogi Tri Prasetyo, Reny Nadlifatin, Satria Fadil Persada, Ma. Janice J. Gumasing

**Affiliations:** 1 School of Industrial Engineering and Engineering Management, Mapúa University, Manila, Philippines; 2 International Bachelor Program in Engineering, Yuan Ze University, Chung-Li, Taiwan; 3 Department of Industrial Engineering and Management, Yuan Ze University, Chung-Li, Taiwan; 4 Department of Information Systems, Institut Teknologi Sepuluh Nopember, Kampus ITS Sukolilo, Surabaya, Indonesia; 5 Entrepreneurship Department, BINUS Business School Undergraduate Program, Bina Nusantara University, Jakarta, Indonesia; ESPOL Polytechnic University, ECUADOR

## Abstract

Palawan is a globally known island located in the Philippines. It has received several recognitions from a variety of international tourism organizations. The study aims to identify the factors affecting continuous visiting behavior to Palawan’s travel accommodations. The study incorporated covariance-based structural equation modeling (CB-SEM) to apply ideologies of Uncertainty Reduction Theory (URT) and Expectation Confirmation Theory (ECT). 712 valid respondents answered an online questionnaire that was distributed to several social media platforms. Structural Equation Modeling (SEM) identified that interactive uncertainty significantly influenced perceived performance compared to passive uncertainty. Both the physical environment and attitude of employees significantly produced an impact on service experience and price acceptance. However, the physical environment negatively influenced price acceptance. Meanwhile, the attitude of employees was the sole exogenous variable that influenced price acceptance. Food and beverage didn’t contribute a significant influence on service experience and price acceptance. Additionally, perceived performance and service experience contributed to positive confirmation. It was also revealed that confirmation was significant towards price acceptance, but was insignificant towards tourist satisfaction. Moreover, price acceptance significantly influenced tourist satisfaction. This study is the first comprehensive study that analyzed the visiting behavior in Palawan. Finally, integrating URT and ECT can be applied and extended, especially for analyzing the visiting behavior of different tourist destinations worldwide.

## 1. Introduction

### 1.1 Background of Palawan Island

Palawan is one of the largest islands in the Philippines. It is a premier destination for local and international tourists [1]. In the years 2013, 2016, 2017, and 2020, Palawan Island was recognized as the “best island in the world” as published by a New York-based international travel magazine [2]. From 2015 to 2019, there was a positive tourist arrival growth rate (16.67% to 29.28%) for Palawan Island, with a total number of visitors ranging from one to two million [3–6]. Hence, the number of tourists unceasingly increased. According to Cahigas et al. [7], tourist satisfaction is highly dependent on positive emotions such as joy, pleasure, and satisfaction. Likewise, Bayih & Singh [8] proved that positive tourist satisfaction contributed to the increasing tourist destination growth rate.

In Puerto Princesa, Palawan, tourists primarily visit the Underground River through a boat ride [4, 9]. Other popular tourist spots are Butterfly Garden, Crocodile Farm, Honda Bay, Sabang Falls, and Mitra’s Ranch, which are included in city tours using land and water transportation modes [4]. Meanwhile, tourists who prefer a variety of water sports and beach hopping tend to visit El Nido, Palawan. It comprised mainstream and remote beaches, lagoons, and lakes. More importantly, snorkeling and scuba diving are the go-to activities of Coron tourists. In Coron, Palawan, ocean creatures and shipwreck views attract tourists.

### 1.2 Research Gap

The study explored Palawan’s travel accommodation through merging Uncertainty Reduction Theory (URT) and Expectation Confirmation Theory (ECT). Travel accommodation offers numerous services to fulfill the tourists’ expectations and preferences, which are both associated with URT and ECT [7, 10]. It pertains to a hotel, guest house, lodge, or any property that suffices as a temporary shelter for tourists. Since all tourists have the authority to post an online review, tourists’ feedback is vital because it reflects their overall experience [11–15].

Although the existing literature applied URT principles to the travel and tourism sectors, the uncertainty strategies failed to identify causes and implications comprehensively. For instance, Buhalis [16] did not support the proposed strategic objectives with appropriate data, including tourists’ real-time feedback. Kusumasondjaja et al. [17] failed to formulate Structural Equation Modeling (SEM)-based significant hypotheses in analyzing international travelers’ behavior in Bali island, Indonesia. While Lee et al. [13] applied URT and SEM to analyze the tourists’ travel accommodation behavior, they focused on the passive method and did not utilize the interactive method.

Moreover, the past studies covered ECT but failed to elaborate on the necessary constructs. One study successfully compared actual experience and expected experience, but the expectation construct was not subdivided into multiple constructs or variables [18]. In addition, Park et al. [19] only considered one expectation construct (travel distance under accessibility or physical environment), other possible constructs were neglected. On the other hand, Filieri et al. [20] grouped travel accommodation attributes (e.g., facilities, cleanliness, food, and location) into one construct, failing to identify the relationship between each attribute and online tourist reviews.

Furthermore, several studies described and analyzed the Philippines’ tourism sector. Following the growing tourism of the Philippines, Maguigad [21], Roxas & Chadee [22], and Valdez et al. [23] recommended environmental and political strategies to improve the Philippines’ tourism campaign. However, these past studies failed to combine quantitative and qualitative approaches in investigating tourists’ insights. Most importantly, there are no existing studies on the factors contributing to tourists’ intention to revisit a travel accommodation in Palawan.

### 1.3 Objective and significance of the study

This study aims to determine the factors affecting tourists’ continuous visiting behavior to Palawan, Philippines, by integrating uncertainty reduction theory (URT) and expectation confirmation theory (ECT). The principles of URT and ECT are integrated through covariance-based structural equation modeling (CB-SEM). Since this study focuses on Palawan’s travel accommodations and tourists’ continuous visiting behavior, it benefits Palawan’s tourism stakeholders economically. The structure of tourism stakeholders involves business owners (including travel accommodations), employees, residents, the government, and tourists.

## 2. Theoretical framework

### 2.1 Travel accommodation in Palawan

Palawan is a globally recognized Philippine island that constantly attracts tourists of all ages, genders, and nationalities [3–6]. Both domestic and international tourists visit the island. Since travel accommodation is a basic necessity for tourists, there is a need to evaluate Palawan’s travel accommodations. Buhalis [16] developed strategic plans to maintain a travel destination’s quality and competitiveness. Other researchers evaluated the Philippines’ tourism by applying statistical and qualitative techniques [21–23]. Due to the lack of relevant studies on Palawan’s travel accommodations, this study aims to create an in-depth analysis of tourists’ insights. It is also believed that positive and negative feedback from tourists influences others’ perceptions of visiting the island’s travel accommodation.

### 2.2 The Determinants of Uncertainty Reduction Theory and Expectation Confirmation Theory

URT refers to people’s tendency to predict a particular behavior due to uncertainties involved in a situation [24]. Unknowingly, people apply this theory to reduce the feeling of disappointment by an undefined behavior. Since a high level of uncertainty causes undefined behavior, people use strategies to seek information from strangers and close contacts to mitigate uncertainties [14]. According to Ramirez et al. [25], people follow interactive, active, extractive, and passive strategies. However, Antheunis et al. [14] highlighted that passive and interactive uncertainty was the most comprehensive strategies. Thus, this study evaluated passive and interactive uncertainty.

Passive uncertainty refers to unnoticeable observation and it’s considered the most efficient method [14, 25]. An example of the passive uncertainty approach is digital media, specifically through online reviews. The online review method is a part of electronic word of mouth (eWOM) because past tourists help future tourists perceive the services offered by a travel accommodation [20]. Future tourists can access information online without exerting excessive physical effort. Hence, this approach was deemed conducive due to its convenience and ability to provide endless information [13]. Therefore, it was hypothesized that:

**Hypothesis 1**. *Passive uncertainty directly influences travel accommodation’s perceived performance*.

Interactive uncertainty emphasizes direct interaction with individuals, hence the most effective method to disclose one’s perceptions [14, 25]. Since interactive uncertainty involves physical contact, tourists can verify the information and ask more questions. Moreover, people tend to disclose important information to information seekers with whom they have established a good relationship [24]. Thus, many tourists seek recommendations from close friends, family, and acquaintances. Lee et al. [13] mentioned that a low level of uncertainty positively impacts a service’s perceived performance. Thus, the following hypothesis was proposed:

**Hypothesis 2**. *Interactive uncertainty directly influences travel accommodation’s perceived performance*.

On the other hand, ECT emphasizes that people’s expectations might lead to confirmation or disconfirmation [26]. Confirmation of expectation equates to positive satisfaction, while disconfirmation of expectation posits negative feedback among the subjects. This study is focused on determining the factors affecting positive confirmation. Sedera et al. [18] revealed that tourists compare their expectations and actual experience. Due to this comparison, the final judgment according to the determining factors is easily depicted by tourists.

Tourists usually expect various services from a travel accommodations [19]. The physical environment of a travel accommodation affects tourists’ expectations because of aesthetics, comfort, and convenience [18]. The physical environment comprises accommodation infrastructure [13, 27], room quality [13, 27], facilities [28], and location [27]. These physical environment factors affect service experience because every tourist has different preferences. Some tourists prefer comfort over aesthetics, while others prefer different physical environment factors. Due to the tourists’ varying preferences, knowing the specific physical environment factor influencing service experience is essential. Past studies also concluded that the physical environment was one of the determining factors of service experience [13, 28]. Hence, this study hypothesized that:

**Hypothesis 3**. *Physical environment directly influences service experience*.

Food and beverage are tangible factors directed to service experience [28]. Food and beverage are part of the tourists’ essentials, which other needs cannot replace. Tourists need to eat and drink at a certain point to replenish the energy they consume every second. Traveling takes tourists’ energy because of the physical activities offered by Palawan travel accommodations. Some activities include swimming, scuba diving, hiking, island hopping, canyoneering, and other sports. In Spain, tourist administrators promoted their travel destinations by focusing on its staple food [12]. Thus, the following hypothesis was proposed:

**Hypothesis 4**. *Food and beverage directly influence service experience*.

Additionally, tourists expect to interact with pleasing employees who they can count on while staying in the travel accommodation [13]. Since travel accommodation is a service industry, its core component is the employees who directly interact with the tourists or guests. Hence, employees must show professionalism and be well-equipped with training and knowledge to develop a positive service experience [13]. Based on past studies, the attitude of employees directly influenced service experience [13, 28, 29]. Through the relevant research, this study hypothesized that:

**Hypothesis 5**. *Attitude of employees directly influences service experience*.

Price is a deal-breaker for tourists since it determines their capability to finance leisure activities. Many tourists evaluate travel accommodation’s cost through the comfort attained from the travel accommodation’s physical environment [30]. As a result, tourists prioritized comfortable accommodation. They accepted the price of travel accommodation when they experienced a relaxing and peaceful room infrastructure [13, 27]. These past studies confirmed that the physical environment produced a direct relationship with price acceptance. Thus, it was hypothesized that:

**Hypothesis 6**. *Physical environment directly influences price acceptance*.

Tourists order food and beverage depending on the taste and corresponding price. In Palawan, every tourist spot is surrounded by restaurants of different classes. It could be a restaurant that offers low-range, mid-range, and high-range services. Thus, tourists can choose among many options that suit their financial capability. Past studies affirmed that food and beverage affect tourists’ price acceptance [31, 32]. However, it was emphasized by El-Adly [30] that food and beverage must be worth their price. Thus, it was hypothesized that:

**Hypothesis 7**. *Food and beverage directly influence price acceptance*.

The attitude of employees reflects the core values of travel accommodation. Employees should show courtesy and professionalism in communicating with tourists. It was emphasized by Lee et al. [13] that employees must receive positive feedback from guests to make them realize the price worthiness of travel accommodation. Other studies also highlighted that travel accommodation employees influenced price acceptance [31, 32]. Many tourists believe that they should be respected and served accordingly. In return, they accept travel accommodation prices wholeheartedly. These past studies affirmed valuable indicators helpful for this research. Thus, the hypothesis is as follows:

**Hypothesis 8**. *Attitude of employees directly influences price acceptance*.

ECT transmits concepts behind the pre- and post-processing of an individual’s decisions [33]. In this study, pre-processing refers to perceived performance. It helps tourists validate predetermined insights and assess actual service experiences. In a nutshell, perceived performance refers to preconceived ideas from online platforms or other people’s feedback. Perceived performance is the tourist’s expectations caused by third-party entities that must be confirmed or disconfirmed [34]. Confirmation or disconfirmation will only happen if a tourist experiences the actual travel accommodation, and it is always important that tourists’ expectations be met or exceeded. Thus, this study hypothesized that:

**Hypothesis 9**. *Perceived performance directly influences confirmation*.

For this study, service experience pertains to the expectations of tourists from the actual services offered by travel accommodations. Tourists typically expect an outstanding and appropriate physical environment, food and beverage, and attitude of employees [13, 18, 27, 28, 30–32]. These three factors are the primary services provided by any travel accommodations. However, people tend to over-expect or under-expect, which affects the confirmation variable [18]. Since result differences were possible, tourists could confirm or disconfirm the travel accommodation’s service experience. Contingent on the past studies, it was hypothesized that:

**Hypothesis 10**. *Service experience directly influences confirmation*.

It was previously discussed that ECT involved post-processing decisions [33]. In the present study, post-processing signifies confirmation because an individual has already finished the evaluation process, specifically to confirm or disconfirm an expectation. Confirmation aims to compare expectations and actual experience. Nevertheless, confirmation is subdivided into three principles. The end result may produce confirmation, positive disconfirmation, and negative disconfirmation [19, 34, 35]. Confirmation denotes that the offered services equally meet tourists’ expectations [34, 35]. Positive disconfirmation occurs once the service experienced by the tourists is deemed to be better than the said expectations [34]. Negative disconfirmation pertains to increased dissatisfaction linked with one’s expectations and actual service experience [33, 34]. Although there are three possible results, this study focuses on confirmation and positive disconfirmation. This study considered the term “confirmation” by merging the concepts of confirmation and positive disconfirmation. The said context is feasible because the tourists’ level of satisfaction is determined proportionally or positively [19, 34]. Moreover, confirmation and positive disconfirmation impact tourist satisfaction, while instigated by impact dissatisfaction [34]. Therefore, the following hypothesis was proposed:

**Hypothesis 11**. *Confirmation directly influences tourist satisfaction*.

Confirmation is the mediating variable between expectations and actual service experience. Once tourists finish the travel accommodation evaluation, they can easily express if their experience is worth its price. However, tourists weigh several factors before accepting the price imposed by a travel accommodation [13]. Most tourists accept the expenses if their expectations exceed or meet the actual service experience. Additionally, past studies confirmed a significant relationship between confirmation and travel destination price [13, 34]. Hence, this study hypothesized the following:

**Hypothesis 12**. *Confirmation directly influences price acceptance*.

Past studies verified that service experience was directly related to tourist satisfaction [13, 27, 28, 30]. In this study, service experience pertains to the expectations of tourists based on the travel accommodation’s physical environment, food and beverage, and the attitude of employees. Furthermore, the service experience engages tourists prior to the actual travel visit, directly influencing tourist satisfaction [36]. Hence, this study hypothesized that the initial expectations of tourists could affect their satisfaction. The following hypothesis was proposed:

**Hypothesis 13**. *Service experience directly influences tourist satisfaction*.

People tend to lay down estimated finance in booking travel accommodation. According to Dogru et al. [32], tourists prioritize lesser travel accommodation costs. Hence, most tourists dislike expensive travel accommodations to mitigate expenses. However, luxury travel accommodation has greater tourist engagement than low-cost travel accommodation [36]. Due to poor marketing, some travel accommodations are neglected. Nonetheless, price acceptance and tourist satisfaction are interconnected variables [8, 13, 19]. Correspondingly, a study concluded that price significantly affected tourist satisfaction [30]. Therefore, this study hypothesized that:

**Hypothesis 14**. *Price acceptance directly influences tourist satisfaction*.

Tourist satisfaction reflects the positive experiences and emotions during the travel period [37]. At the end of every travel period, tourists reflect if they feel satisfied or dissatisfied with their overall experience in the travel accommodation. Thus, tourist satisfaction is a crucial indicator of positive feedback toward travel accommodation. Many studies emphasized that positive tourist satisfaction often leads to continuous visiting behavior [18, 37, 38]. Tourists’ continuous visiting behavior characterizes tourist loyalty and their intention to revisit the travel accommodation. Sedera et al. [18] emphasized that tourists’ continuous visiting behavior is dependent on their level of satisfaction. If tourists are pleased with the travel accommodation services and actual experiences, they are expected to revisit the same travel accommodation. It was also proven by Pestana et al. [37] that tourists’ motivations and emotions affect their continuous visiting behavior. However, dissatisfied tourists tend to look for better travel accommodations. Furthermore, the studies of El-Adly [30] and Hung et al. [38] verified that tourist satisfaction significantly influenced continuous visiting behavior. Additionally, Lee et al. [13] itemized that online experience contributed to tourist satisfaction, eventually leading to tourists’ continuous visiting behavior. Hence, the subsequent hypothesis was proposed:

**Hypothesis 15**. *Tourist satisfaction directly influences continuous visiting behavior*.

**Fig 1:**
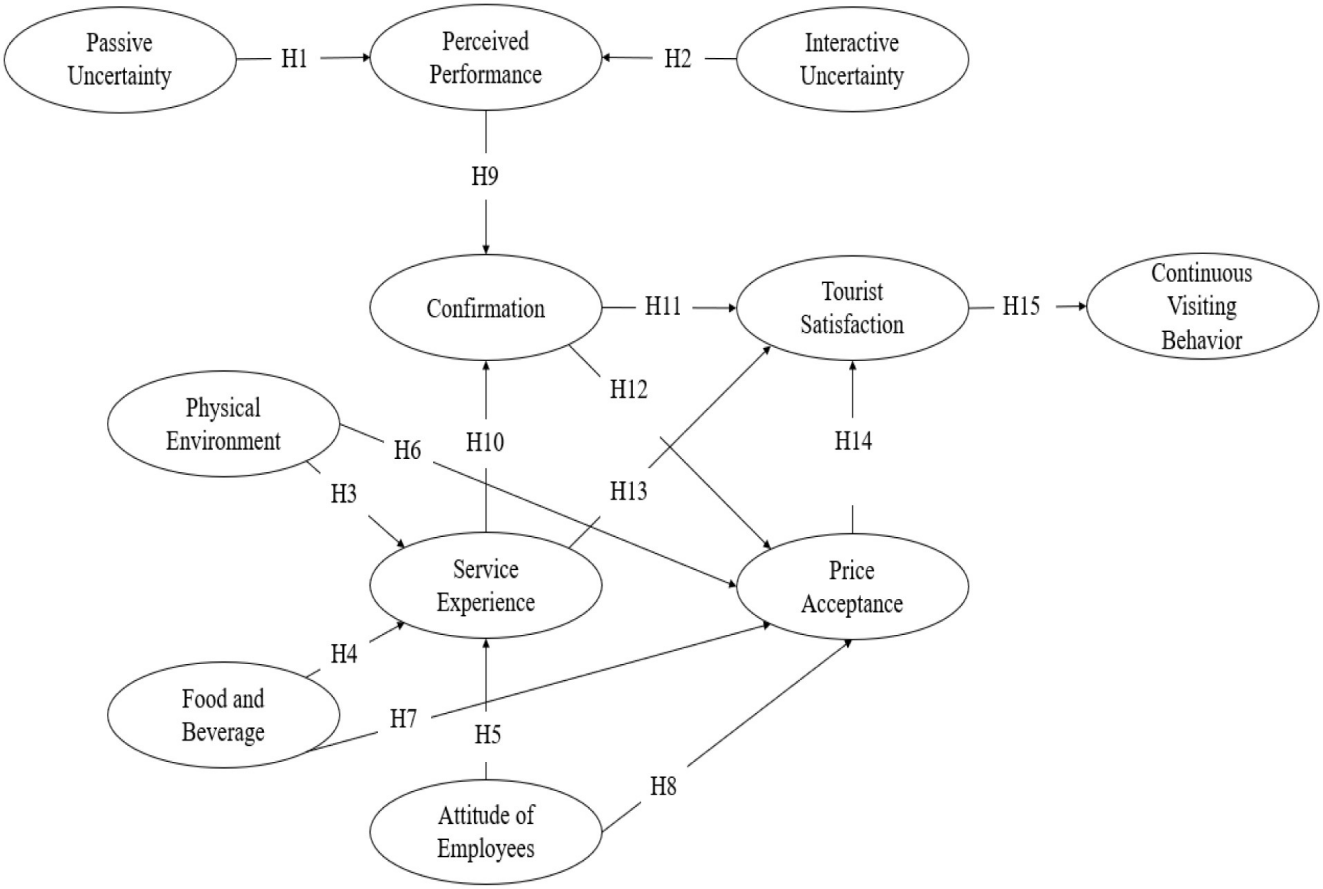
Initial theoretical framework.

Numerous studies hypothesized and proved theoretical models to identify factors affecting tourist satisfaction and continuous visiting behavior. Lee et al. [13] incorporated URT to analyze tourists’ insights between the online experience and the actual service experience. Tourists tend to doubt new services introduced to them. As a result, people seek information through different communication mediums and online platforms to reduce uncertainty [20]. On the other hand, ECT is commonly applied to service quality, tourist satisfaction, and continuous visiting behavior [34, 35]. According to the theory, tourists are satisfied if their expectations meet or exceed the actual service they received. The study of Chen et al. [33] resulted in a comprehensive understanding of ECT based on future behavioral intentions and prior travel accommodation experience. Fig 1 shows the proposed framework combining URT and ECT to identify the determinants affecting tourist’s continuous visiting behavior.

## 3. Methodology

This study was approved by Mapua University Research Ethics Committee and in accordance with Data Privacy Act or Republic Act No. 10173 in the Philippines. Prior to the data collection, all participants signed a consent form that informed them about the purpose of the study and the procedure.

### 3.1 Research paradigm and sampling approach

The researchers applied the pragmatism research paradigm. This research paradigm focuses on the problem, allows method customization, and explores the best technique dependent on the problem (Rahi, 2017). Pragmatism was utilized because the study’s research framework was a combination of two existing theories (URT and ECT). These theories were merged through the support of past studies and were tested using the present study. Furthermore, URT and ECT were modified by adding and removing latent variables based on supporting quantitative and qualitative studies. It was also noted that pragmatism was a combination of positivist and interpretive paradigms (Rahi, 2017). Whereby, the researchers verified old theories, generated a new framework, and applied experimental and social approaches.

Moreover, the researchers executed a non-probability convenience sampling approach. This sampling approach allows efficient data collection and eliminates time-wasting tasks (Rahi, 2017). By the time researchers conducted the study, the number of COVID-19 cases kept increasing in the Philippines. This scenario prohibited researchers to explore other avenues to distribute the questionnaire. Thus, the researchers maximized online messaging platforms to reach out to the current network, ask for their consent to participate in the study, and encourage people to answer the survey. This approach produced good results as the researchers gathered 712 valid respondents in 3 months.

### 3.2 Participants

Google Forms, a web-based survey tool, was utilized to collect information and responses from participants. The questionnaire was created through Google Forms and it was disseminated through online platforms like Facebook, Messenger, and Instagram. These social media websites were maximized because they were included in the Philippines’ top 5 most used applications [39]. Then, the participant’s responses were automatically submitted to the researchers’ primary Google Forms file and extracted into an Excel file. As a result, the study accumulated 777 Filipino participants from January to March 2021. However, 65 participants had no intention to visit Palawan, had never visited any travel accommodations, and uttered irrelevant comments on data quality checking approaches. Thus, the study accumulated a total of 712 valid survey responses. Participants who possess this attitude and experience most likely have different interpretations than those who have visited Palawan and stayed in travel accommodations. Consequently, the purpose of eliminating unnecessary responses was to meet the study’s objective, which was to conceptualize the tourists’ continuous visiting behavior in visiting Palawan’s travel accommodation. Additionally, this study applied Yamane Slovin’s formula to find an acceptable research sample size. The past studies recommended that 5% error is the optimal solution and at least 399 valid responses are the recommended sample size [40, 41]. Since this study accumulated 712 valid survey responses, the number of respondents used was acceptable.

Table 1 shows the different characteristics of the participants. Valid survey responses accumulated a total of 712 Filipino participants. Specifically, the study gathered more female participants (75.84%) than male participants (24.16%). The common age range of participants was between 18–24 years old (51.54%), followed by 25–34 years old (23.74%), ≤ 17 years old (12.22%), 35–44 years old (6.46%), 45–54 years old (3.93%), and, lastly ≥ 55 years old (2.11%). Moreover, most of the participants were students (45.79%), followed by full-time employees (29.63%), then unemployed (14.47%), self-employed (6.18%), and employed part-time (3.93%). Before the COVID-19 pandemic, participants visited Palawan 1 to 2 times every year (64.61%) and they would spend an average of 1 to 2 days in the travel accommodation. Only a few participants preferred to travel to Palawan at least 3 times a year and stay in travel accommodation for at least 3 days. Finally, the participants’ most preferred travel accommodation budget was the least among the choices, amounting to ≤ ₱1,000 (25.98%).

**Table 1:** Demographic profile of participants.

Characteristic	Category	N	%
Gender	Male	172	24.16%
	Female	540	75.84%
Age	≤ 17 years old	87	12.22%
	18–24 years old	367	51.54%
	25–34 years old	169	23.74%
	35–44 years old	46	6.46%
	45–54 years old	28	3.93%
	≥ 55 years old	15	2.11%
Employment status	Student	326	45.79%
	Unemployed	103	14.47%
	Part-time employment	28	3.93%
	Self-employed	44	6.18%
	Full-time employment	211	29.63%
Travel frequency	1–2 times a year	460	64.61%
	3–4 times a year	153	21.49%
	5–6 times a year	40	5.62%
	6–7 times a year	13	1.83%
	≥ 8 times a year	46	6.46%
Average length of trip	1–2 days	488	68.54%
	3–4 days	198	27.81%
	≥ 5 days	26	3.65%
Travel accommodation budget	≤ ₱1,000	185	25.98%
	₱1,001-₱2,000	162	22.75%
	₱2,001-₱3,000	111	15.59%
	₱3,001-₱4,000	131	18.40%
	₱4,001-₱5,000	49	6.88%
	≥ ₱5,001	74	10.39%

### 3.3 Questionnaire

The questionnaire was subdivided into two sections: (1) demographic profile of participants; and (2) tourist satisfaction with travel accommodation. The first part consists of the participants’ demographic characteristics. It comprised gender, age, employment status, travel frequency, average length of stay in the travel accommodation, and budget for travel accommodation. In the second part of the questionnaire, a 5-point Likert scale was utilized to determine the extent of tourists’ insights on travel accommodation (Table 2). The questionnaire’s term “travel accommodation” refers to any hotels/lodges/guesthouses that participants have experienced.

**Table 2:** Constructed questionnaire for structural equation modeling.

Construct	Item	Measure	Reference
Passive Uncertainty	PU1	Information on travel accommodation’s social media websites was helpful.	Lee et al. [13]
	PU2	Online reviews posted by travel accommodation guests affect my decision.	
	PU3	I often look on the internet to help me decide before booking the travel accommodation.	Lee et al. [13]
	PU4	Social media sites provide useful information about travel accommodation.	Chen et al. [33]
Interactive Uncertainty	IU1	I usually ask my friends and family about their recommended travel accommodations every time I go on vacation.	Chen et al. [33]
	IU2	My decision in booking travel accommodation depends on friends’ and family’s feedback.	Chen et al. [33]
	IU3	I often recall past recommendations from friends and family when booking travel accommodation.	
	IU4	I usually let other people decide for me though I ask others for recommendations	
Perceived Performance	PP1	I feel that the travel accommodation’s reputation is generally reliable based on online reviews.	Chen et al. [33]
	PP2	I feel that the travel accommodation will meet my expectation based on friends’ and family’s feedback.	Chen et al. [33]
	PP3	I feel that the information I gathered about the travel accommodation is trustworthy.	Chen et al. [33]
	PP4	I feel that the place is quite close to my ideal travel accommodation.	Nunkoo et al. [27]
	PP5	I feel that I will be satisfied with my choice of travel accommodation.	
Confirmation	C1	The travel accommodation kept promises and commitment to the services they offered.	Chen et al. [33]
	C2	The travel accommodation laid out important information about their amenities.	Chen et al. [33]
	C3	The travel accommodation provided accurate visual representations.	Chen et al. [33]
	C4	I always get a remarkable experience every time I visit the travel accommodation.	
Physical Environment	PE1	I expect the travel accommodation’s room size to be adequate.	Nunkoo et al. [27]
	PE2	I expect the travel accommodation’s interior design to be aesthetically pleasing.	Lee et al. [13]; Nunkoo et al. [27]
	PE3	I expect the travel accommodation to be clean.	Lee et al. [13]; Nunkoo et al. [27]
	PE4	I expect the travel accommodation’s bed, mattress, and pillow to be comfortable.	Nunkoo et al. [27]
	PE5	I expect the travel accommodation’s bath amenities and air conditioning to be appropriate.	Al-Refaie [28]
	PE6	I expect the travel accommodation’s physical facilities to be visually attractive (e.g., spa, gym, and swimming pool).	Al-Refaie [28]
	PE7	I expect the travel accommodation to be accessible by public transport.	Nunkoo et al. [27]
	PE8	I expect the travel accommodation to provide adequate security features.	Nunkoo et al. [27]
	PE9	I expect the travel accommodation’s internet connection to be accessible and stable.	
Food and Beverage	FB1	I expect that the travel accommodation provides a variety of food and beverage services that I can choose from.	Nunkoo et al. [27]; Al-Refaie [28]
	FB2	I expect that the travel accommodation prepares and deliver food and beverage on time.	Nunkoo et al. [27]; Al-Refaie [28]
	FB3	I expect that the travel accommodation provides luxurious dishes.	
	FB4	I expect that the travel accommodation provides quality food and beverage.	Al-Refaie [28]
Attitude of Employees	AE1	I expect that the travel accommodation’s employees are courteous and professional.	Al-Refaie [28]
	AE2	I expect that the travel accommodation’s employees demonstrate their willingness to help me.	Nunkoo et al. [27]; Al-Refaie [28]
	AE3	I expect that the travel accommodation employees can solve my problems.	Nunkoo et al. [27]; Al-Refaie [28]
	AE4	I expect that the travel accommodation’s employees provide a thorough and satisfactory service.	Lee et al. [13]
Service Experience	SE1	I expect adequate and quality travel accommodation room.	Nunkoo et al. [27]; Al-Refaie [28]
	SE2	I expect high-quality travel accommodation infrastructure.	Nunkoo et al. [27]
	SE3	I expect physical facilities and amenities provided by the travel accommodation.	Nunkoo et al. [27]
	SE4	I expected an ideal location for travel accommodation.	Al-Refaie [28]
	SE5	I expect safety & security features from the travel accommodation.	Nunkoo et al. [27]
	SE6	I expect to trust the behavior of the employees who provide services in the travel accommodation.	Nunkoo et al. [27]; & Al-Refaie [28]
	SE7	I expect high-quality food and beverage from the travel accommodations.	Nunkoo et al. [27]; Al-Refaie [28]
Price Acceptance	PA1	I consider my staying experience fortunate through bargains (e.g., special rates, offers, and discounts).	El-Adly [30]
	PA2	I am willing to pay for excellent travel accommodation services.	Lee et al. [13]
	PA3	I am willing to pay for additional charges.	Lee et al. [13]
	PA4	The food and beverage served at travel accommodations are worth their prices.	El-Adly [30]
	PA5	The travel accommodations provide basic service (e.g., housekeeping and room service) worth its price.	El-Adly [30]
	PA6	The travel accommodations offer worthy amenities (e.g., spa and gym).	El-Adly [30]
	PA7	The travel accommodations’ services and prices complement each other.	Lee et al. [13]
Tourist Satisfaction	TS1	I feel satisfied with the travel accommodation’s overall service.	Lee et al. [13]; Nunkoo et al. [27]; Hung et al. [38]
	TS2	I feel satisfied with the accuracy of my research and the positive end-result of my travel accommodation experience.	Lee et al. [13]; Nunkoo et al. [27]
	TS3	I find it worthy spending time researching travel accommodation because it generated excellent results.	González-Mansilla et al. [15]
	TS4	I feel satisfied with the travel accommodation’s price.	González-Mansilla et al. [15]; El-Adly [30]
Continuous Visiting Behavior	CVB1	I intend to revisit the travel accommodation I went to when traveling to Palawan.	Lee et al. [13]; Chen et al. [33]
	CVB2	I intend to prioritize the travel accommodation I went to compared to other accommodations when traveling to Palawan.	Lee et al. [13]; Chen et al. [33]
	CVB3	I will make an effort to book the same travel accommodation when traveling to Palawan.	Lee et al. [13]; Chen et al. [33]
	CVB4	I intend to be a loyal customer of the travel accommodation I went to when traveling to Palawan.	Chen et al. [33]; Hung et al. [38]

The researchers utilized data quality protocols when distributing the questionnaire and filtering valid responses. These techniques ensured the reliability and credibility of respondents’ answers. As a result of these data quality approaches, a total of 65 respondents were eliminated because of their responses’ irrelevance.

The first protocol used by researchers was showcasing questions about each construct on one page, resulting in a total of eleven subdivided pages. All pages have corresponding guidelines to ensure that respondents could follow the given questions. They were also constantly reminded by including a big and bold note to read each question carefully.

This was further strengthened by including an open-ended mandated question that asked respondents about their personal opinions on random constructs. Their responses were checked by researchers individually and irrelevant responses were eliminated from the set of valid responses. For instance, one respondent mentioned that tourist satisfaction pertains to employees’ hospitality. Unfortunately, this was deemed incorrect because the respondent’s explanation was more associated with the Attitude of Employees construct. Another instance was when a participant copy-pasted a similar answer across all open-ended boxes.

Another screening technique was the inclusion of red herring questions. These were implemented to ensure the quality of responses by placing random questions on different pages. An example of a red herring question under the Physical Environment construct page was when participants were asked, “Which city of Palawan best describes your ideal physical environment?” One of the respondents answered a city that was not included in Palawan, and hence was eliminated from valid responses. At some points, participants were also asked to rank their preferences for visiting popular cities like Puerto Princesa, Coron, and El Nido; whereby 1 refers to their topmost preference and 3 is the least preference. It was considered a tricky question since they should they were instructed to rank each city from 1 to 3 and similar rankings were not allowed. Thus, participants who answered with a similar set of rankings were removed from the study.

Furthermore, common method bias and other biases were addressed by employing Fornell-Larkcer and Heterotrait-monotrait ratio. These two methods ensured reliable findings through cross-sectional construct checking and discriminant validity technique. They employed statistical techniques that could determine the relationship accuracy between targeted constructs and participants’ responses.

### 3.4 Covariance-Based Structural Equation Modeling

Structural Equation Modeling (SEM) is a comprehensive multivariate statistical approach that determines interrelationships between multiple variables [42]. SEM utilizes fundamental theories to develop a conceptual framework. It also defines comprehensive results behind the complex data and inputs. Most importantly, the study applied the covariance-based structural equation modeling (CB-SEM) approach. The CB-SEM approach is focused on the theoretical covariance matrix because it aims to evaluate the identified theories [43]. Since this study aims to integrate URT and ECT, the developed model is complex yet comprehensive. CB-SEM is the most appropriate statistical technique to evaluate integrated theories. In addition, this approach also determines the relationships between the identified constructs, especially in analyzing the constructs affecting tourists’ continuous visiting behavior. SPSS 22 and Amos 22 were the software used to generate significant results from the constructed CB-SEM.

Factor loading (FL), Cronbach’s alpha (α), composite reliability (CR), and average variance extracted (AVE) measure the validity of data, constructs, and measures [42]. Hence, FL, α, CR, and AVE were calculated. First, FL must be at least 0.50 to indicate a reliable measure [42]. Second, the recommended value for α is at least 0.70 to ensure internal consistency [44]. Third, CR should be at least 0.70 to signify data consistency [42]. Lastly, the suggested value for AVE is 0.50 to indicate lesser errors in measures than the variance in constructs [42]. However, this study applied the findings of Fornell & Larcker [45], where the AVE of less than 0.50 is acceptable if the CR is higher than 0.60.

After the validity of data, constructs, and measures was confirmed, CB-SEM fit indices were calculated. The study produced CB-SEM fit indices integrated with the maximum likelihood approach to generate the necessary results. CB-SEM fit indices were segmented into Incremental Fit Index (IFI), Tucker Lewis Index (TLI), and Comparative Fit Index (CFI) [42, 46, 47]. Additionally, the goodness of fit was measured through Goodness of Fit Index (GFI) and Adjusted Goodness of Fit Index (AGFI) [42, 46, 47]. A good model must generate a value greater than 0.80 for all the aforementioned indices [48]. For the badness of fit, Root Mean Square Error of Approximation (RMSEA), Root Mean squared Residual (RMR), and Standardized Root Mean squared Residual (SRMR) recommended values were less than 0.07, 0.08, and 0.08, respectively, to conclude a good fit between the model and the data [42, 46, 47, 49]. Finally, the chi-squared value divided by the degrees of freedom (CMIN/df) was generated to act as support for the overall model. Its recommended value should be less than or equal to 5.00 [50].

## 4. Results

The researchers tested the data normality before the application of CB-SEM. They utilized Shapiro-Wilk’s Normality z-score and the current data passed the threshold within ± 1.96. This standard procedure and value were supported by another study [51].

**Fig 2:**
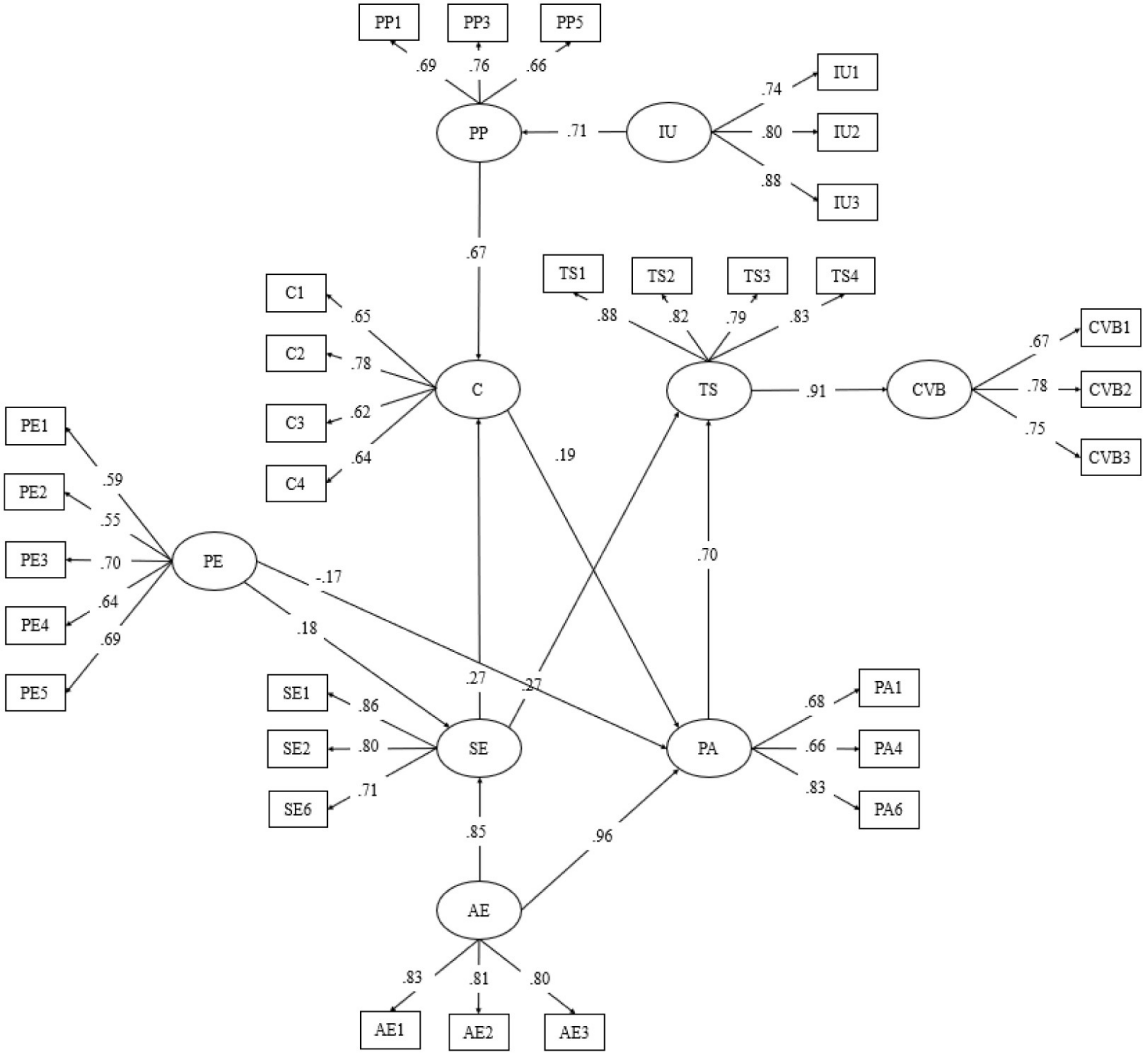
Final theoretical framework. IU: Interactive Uncertainty; PP: Perceived Performance; C: Confirmation; PE: Physical Environment; AE: Attitude of Employees; SE: Service Experience; PA: Price Acceptance; TS: Tourist Satisfaction; CVB: Continuous Visiting Behavior.

The initial model presented in Fig 2 was further improved by eliminating insignificant constructs and measures. First, all four measures under passive uncertainty produced a factor loading of less than 0.50. Hence, the passive uncertainty construct was removed due to weak factor loading. Hair et al. (2010) indicated that 0.50 is the minimum value needed for factor loading. Second, food and beverage were not significant (p-value > 0.05) to service experience (H4) and price acceptance (H7), therefore H4 and H7 were discarded. Since the food and beverage construct was insignificant to the interlinked hypotheses, this construct was removed. Third, H11 was removed from the model because confirmation didn’t directly influence tourist satisfaction (p-value > 0.05). As a result, Fig 2 reflects the final model generated through Amos 22.

Table 3 shows the corresponding CB-SEM results based on the final theoretical framework. Factor loadings associated with each measure passed the minimum cut-off of 0.50, implying that measures strongly support the interlinked hypotheses [42]. The Cronbach’s alpha (α) for all constructs exceeded the suggested value of 0.70 which indicated high internal consistency and reliability [28, 30, 44]. The composite reliability (CR) for all constructs passed the suggested cut-off of 0.70, signifying internal measurements’ consistency [27, 30, 42]. Furthermore, the average variance extracted (AVE) of interactive uncertainty (IU), perceived performance (PP), attitude of employees (AE), service experience (SE), price acceptance (PA), tourist satisfaction (TS), and continuous visiting behavior (CVB) passed the suggested value of 0.50. Constructs with at least 0.50 AVE values implied lesser construct errors [27, 30, 42]. However, the AVE of confirmation (C) and physical environment (PE) fell below 0.50. This instance occurred due to the constructs’ respective factor loadings, which had high proximity to the minimum value of 0.50. Nonetheless, Fornell & Larcker [45] indicated that the convergent validity of constructs is acceptable despite not attaining a minimum value of AVE if the Cronbach’s Reliability (CR) is at least 0.60.

**Table 3:** Results of structural equation modeling.

Construct	Measure	FL	Cronbach’s α	CR	AVE
Interactive Uncertainty	IU1	0.74	0.87	0.85	0.65
	IU2	0.80			
	IU3	0.88			
Perceived Performance	PP1	0.69	0.74	0.75	0.50
	PP3	0.76			
	PP5	0.66			
Confirmation	C1	0.65	0.74	0.77	0.46
	C2	0.78			
	C3	0.62			
	C4	0.64			
Physical Environment	PE1	0.59	0.73	0.73	0.40
	PE2	0.55			
	PE3	0.70			
	PE5	0.69			
Attitude of Employees	AE1	0.83	0.87	0.85	0.66
	AE2	0.81			
	AE3	0.80			
Service Experience	SE1	0.86	0.85	0.83	0.62
	SE2	0.80			
	SE6	0.71			
Price Acceptance	PA1	0.68	0.74	0.77	0.52
	PA4	0.66			
	PA6	0.83			
Tourist Satisfaction	TS1	0.88	0.91	0.90	0.69
	TS2	0.82			
	TS3	0.79			
	TS4	0.83			
Continuous Visiting Behavior	CVB1	0.67	0.79	0.78	0.54
	CVB2	0.78			
	CVB3	0.75			

In addition, all qualified measures from the final CB-SEM model underwent skewness and kurtosis statistical processes. Based on a relevant study, both skewness and kurtosis should have values within the range of -2.00 to +2.00 [52]. As seen in Table 4, all values met the required estimates, which supported the regularity of data distribution. Specifically, the measures’ skewness varied from -1.383 to -0.380 and kurtosis ranged from -0.933 to 1.574.

**Table 4:** Skewness and kurtosis values.

Measure	Skewness	Kurtosis	Measure	Skewness	Kurtosis
IU1	-1.254	.783	AE2	-1.166	.843
IU2	-1.051	.271	AE3	-.905	.118
IU3	-1.051	.566	SE1	-.944	-.141
PP1	-1.148	1.178	SE2	-1.111	.887
PP3	-1.059	1.574	SE6	-1.199	.844
PP5	-.777	-.521	PA1	-1.316	1.461
C1	-.960	.726	PA4	-.883	.116
C2	-1.087	.584	PA6	-.701	-.891
C3	-1.097	1.083	TS1	-1.059	.492
C4	-1.164	1.161	TS2	-.867	-.131
PE1	-.522	-.605	TS3	-.882	-.041
PE2	-.380	-.933	TS4	-1.044	.808
PE3	-1.383	1.200	CVB1	-1.298	.540
PE5	-1.199	.599	CVB2	-1.193	.525
AE1	-1.076	.274	CVB3	-.837	-.367

As seen in Table 5, the p-values of constructs with a direct relationship were statistically significant. Past studies concluded that a significant p-value is less than or equal to 0.05 [42, 46, 47]. Among the 11 directly connected constructs, 8 hypotheses (H2, H3, H5, H9, H10, H12, H14, H15) had a p-value of 0.001, while the remaining 3 hypotheses (H6, H8, H13) produced p-values of 0.012, 0.040, and 0.048, respectively. Nonetheless, all p-values met the minimum cut-off of 0.05. Aside from p-values, Table 5 also distinguishes the equivalent total effect, direct effect, and indirect effect for each interconnected construct. The total effect is the sum of direct effect and indirect effect values.

**Table 5:** The p-value and corresponding effects associated with significant constructs.

Connected Construct	P-value	Total Effect	Direct Effect	Indirect Effect
H2: IU → PP	0.001	0.71	0.71	-
IU → C	0.372	0.48	-	0.48
IU → PA	0.065	0.09	-	0.09
IU → TS	0.058	0.07	-	0.07
IU → CVB	0.034	0.06	-	0.06
H3: PE → SE	0.001	0.18	0.18	-
PE → C	0.065	0.05	-	0.05
H5: AE → SE	0.001	0.85	0.85	-
AE → C	0.228	0.23	-	0.23
H6: PE → PA	0.012	-0.16	-0.17	0.01
PE → TS	-0.098	-0.06	-	-0.06
PE → CVB	-0.057	-0.06	-	-0.06
H8: AE → PA	0.04	1.01	0.96	0.04
AE → TS	0.903	0.93	-	0.93
AE → CVB	0.62	0.85	-	0.85
H9: PP → C	0.001	0.68	0.68	-
PP → PA	0.114	0.13	-	0.13
H10: SE → C	0.001	0.27	0.27	-
SE → PA	0.054	0.05	-	0.05
PP → TS	0.102	0.09	-	0.09
PP → CVB	0.059	0.08	-	0.08
H12: C → PA	0.001	0.19	0.19	-
C → TS	0.157	0.14	-	0.14
C → CVB	0.091	0.12	-	0.12
H13: SE → TS	0.048	0.30	0.27	0.04
SE → CVB	0.231	0.28	-	0.28
H14: PA → TS	0.001	0.70	0.70	-
PA → CVB	0.519	0.64	-	0.64
H15: TS → CVB	0.001	0.91	0.91	-

The final CB-SEM model fit was analyzed through the indices presented in Table 6. The values under IFI, TLI, CFI, GFI, and AGFI exceeded the suggested value of 0.80. Additionally, RMSEA was 0.087, which had 80.46% proximity to the cut-off. Since the RMSEA value was near the suggested cut-off, RMSEA value was accepted [48]. In addition, the model’s RMR and SRMR were 0.051 and 0.072, respectively, which further proved that the model fit indices were undeniably acceptable [42, 48]. Furthermore, CMIN/df value produced a reasonable fit at 4.594. Lastly, the overall model’s p-value was extremely significant at a 0.001 significance level. Therefore, the data was concluded as a perfect fit for the final model.

**Table 6:** The model fit indices for structural equation modeling.

SEM Goodness of Fit Measure	Parameter Estimate	Suggested Cut-off	Reference
Incremental Fit Index (IFI)	0.86	> 0.80	Gefen et al. [48]
Tucker Lewis Index (TLI)	0.84	> 0.80	Gefen et al. [48]
Comparative Fit Index (CFI)	0.86	> 0.80	Gefen et al. [48]
Goodness of Fit Index (GFI)	0.83	> 0.80	Gefen et al. [48]
Adjusted Goodness of Fit Index (AGFI)	0.81	> 0.80	Gefen et al. [48]
Root Mean Square Error of Approximation (RMSEA)	0.087	< 0.07	Hair et al. [42]
Root Mean Square Residual (RMR)	0.051	< 0.08	Hair et al. [42]
Standardized Root Mean squared Residual (SRMR)	0.072	< 0.08	Hair et al. [42]
Chi-squared (χ2 or CMIN)	1722.717	No standard (data-dependent)	Marsh & Hocevar [50]
Degrees of freedom (df)	375	No standard (data-dependent)	Hair et al. [42]
χ2/df	4.594	≤ 5.00	Marsh & Hocevar [50]
P-value	0.001	≤ 0.05	Hair et al. [42]

A criterion analysis named Fornell-Larkcer was employed to ensure that the correlations among latent variables were unbiased [51]. According to a past study, the diagonal values of cross-sectional constructs should be greater than the off-diagonal values [51]. It could be depicted from Table 7 that the cross-sectional constructs had the highest values (0.892, 0.753, 0.84, 0.895, 0.817, 0.747, 0.815, 0.877, 0.885) in their corresponding columns. Thus, the discriminant validity of nine latent variables was supported by comparing constructs’ variances.

**Table 7:** Fornell-Larcker criterion analysis.

	AE	C	CVB	IU	PA	PE	PP	SE	TS
AE	0.892								
C	0.531	0.753							
CVB	0.668	0.444	0.84						
IU	0.333	0.444	0.323	0.895					
PA	0.753	0.549	0.686	0.358	0.817				
PE	0.595	0.562	0.499	0.44	0.515	0.747			
PP	0.551	0.662	0.491	0.518	0.543	0.578	0.815		
SE	0.804	0.479	0.727	0.376	0.754	0.62	0.545	0.877	
TS	0.791	0.531	0.758	0.389	0.804	0.576	0.564	0.774	0.885

Following the previous method, another procedure that supported the mitigation of common method biases is called the Heterotrait-monotrait (HTMT) ratio. This technique was commonly used in SEM by calculating correlation estimates among constructs and values should be less than 0.90 [51]. Table 8 displays the corresponding values ranging from 0.188 to 0.873 and all of them passed the required parameter value. Hence, all constructs were unique and aligned with the discriminant validity of the proposed theory.

**Table 8:** Heterotrait-monotrait ratio.

	AE	C	CVB	IU	PA	PE	PP	SE	TS
AE									
C	0.617								
CVB	0.536	0.414							
IU	0.384	0.54	0.188						
PA	0.739	0.658	0.548	0.387					
PE	0.653	0.769	0.412	0.552	0.604				
PP	0.63	0.873	0.426	0.629	0.556	0.781			
SE	0.857	0.523	0.608	0.417	0.713	0.672	0.641		
TS	0.843	0.638	0.581	0.432	0.839	0.688	0.686	0.849	

## 5. Discussion

### 5.1 Interpretation of results

This subsection itemized the importance and nonsignificance of each hypothesis. Subsection 5.1 was categorized and chronologically organized based on direct connections associated with latent variables. Moreover, the hypotheses comparison to supporting studies and arguments of the present study were explained.

#### 5.1.1 Hypothesis 1 and 2 analysis

The principles behind H1 and H2 were formulated through URT, and URT is one of the theories applied to this study. URT emphasizes passive and interactive methods to reduce uncertainties before tourists travel to the accommodation place [13, 14, 25]. These past studies considered hypothetical scenarios prior to visiting the travel destination place.

However, the present study rejected H1 because of the insignificant p-value (p > 0.50) and the factor loadings of passive uncertainty were unacceptable (FL > 0.50). Although Lee et al. [13] stated that passive uncertainty or online experience was the most common method because of its convenience, this current research proved that passive uncertainty was ineffective in evaluating the perceived performance of travel accommodations. Alonso-Almeida et al. [12] also stated that online platforms are dangerous because some tourists promote a travel destination without considering the risks and security.

Meanwhile, H2 was deemed significant because interactive uncertainty directly influenced perceived performance (β:0.71, p:0.001). Hence, this study concluded that direct interaction (interactive uncertainty) is the most significant method in determining the perceived performance of travel accommodation than searching online reviews (passive uncertainty). Tourists trusted the insights of personal acquaintances, family, and friends compared to strangers. Similar results were found in the study of Antheunis et al. [14], concluding that interactive uncertainty had the strongest correlation among the uncertainty reduction strategies. Ramirez et al. [25] also proved that direct communication was advantageous to the information seeker. In this study, the information seeker signifies the tourists who participated in the questionnaire, while the information giver pertains to the acquaintances of the tourists. Since the information seeker controls the communication process, the information giver is enticed to disclose crucial information such as affective opinions and personal experiences. Moreover, tourists were more comfortable asking personal questions to their acquaintances, family, and friends. Thus, they could freely ask for and receive the information that they wanted.

#### 5.1.2 Hypothesis 3 to 8 analysis

According to Chen et al. [33], the top three service quality dimensions were physical environment, food and beverage, and attitude of employees, in sorted order of highest to lowest rank. This study hypothesized that the three service quality dimensions (physical environment, food and beverage, and attitude of employees) directly influenced service experience (H3, H4, and H5) and price acceptance (H6, H7, and H8). Among these hypotheses, H3, H5, H6, and H8 were significant (p-value **≤** 0.05), while H4 and H7 were found insignificant (p-value > 0.05). The succeeding paragraphs tackled H3 and H6 simultaneously, followed by H5 and H8 combination, and finally the non-significant H4 and H7.

Although food and beverage were insignificant, the study proved that tourists prioritized the physical environment and attitude of employees. The physical environment significantly positively service experience (H3: β:0.18, p:0.001) but negatively influenced price acceptance (H6: β:-0.17, p:0.012). This result implied that tourists expect the physical environment of travel accommodations to be attractive, convenient, and comfortable. Specifically, the participants were asked about their insights on accommodation infrastructure, aesthetics, cleanliness, and amenities. In a similar study, services offered by travel accommodations (e.g., amenities, aesthetics, customer service) positively influenced tourists’ service experiences [28]. In other studies, the physical environment was insignificant to positive service experience [13, 30]. The results of this study also implied tourists are unforgiving in price/cost because they do not see the worth of paying a hefty amount for the physical environment.

Additionally, the attitude of employees positively influenced service experience (H5: β:0.85, p:0.001) and price acceptance (H8: β:0.96, p:0.040). Tourists expect the travel accommodation’s employees to be professional, hospitable, and well-equipped. They depict positive service experiences if the employees serve them well. Consequently, the price worthiness of Palawan’s travel accommodation depends on employees’ customer service skills. Filipinos are hospitable by nature and this trait might lead to a positive tourist experience [53]. Contrary to the study of El-Adly [30], the attitude of employees didn’t produce a remarkable influence on service experience. This discrepancy is possible because the study of El-Adly [30] is engrossed in UAE travel accommodations, while this study focuses on Palawan, Philippines. One study also revealed that the attitude of employees did not produce any significant influences on price acceptance [13]. Differences in the results are possible because Lee et al. [13] focused on Korean perceptions and participants’ demographic profile characteristics were entirely different.

H4 and H7 were the hypotheses formulated from the food and beverage construct. Although past studies verified the importance of food and beverage in travel accommodations [30, 33], this study proved that food and beverage were not significant towards service experience and price acceptance. Likewise, Nunkoo et al. [27] rejected the direct influence of food and beverage on tourist satisfaction. Food and beverage were considered physiological needs, but this construct didn’t necessarily affect tourists’ perceptions. Since this construct is a part of ECT, tourists do not expect a particular food and beverage. Thus, any kind of food and beverage served on the table would not create a big impact on tourists’ expectations.

#### 5.1.3 Hypothesis 9 and 10 analysis

H9 and H10 were supported. Both perceived performance (H9: β:0.68, p:0.001) and service experience (H10: β:0.27, p:0.001) produced a significant and positive influence on confirmation. The relationship between these constructs generated positive confirmation, which indicated that tourists’ expectations were met or exceeded. Perceived performance signified reduced uncertainty from interactive method and service experience denoted tourists’ expectations. Tourists showed eagerness to validate their assumptions about Palawan’s travel accommodations. This result confirmed that Palawan travel accommodation’s truthful and accurate information significantly impacted the tourist’s judgment. Similar to the study of Fu et al. [35], perceived performance and service quality significantly influenced confirmation.

#### 5.1.4 Hypothesis 11 to 13 analysis

Next, H11 was rejected because confirmation didn’t significantly influence tourist satisfaction (p-value > 0.05). This result emphasized that the confirmed expectations of tourists did not contribute an immense effect on their satisfaction. Tourists might or might not feel satisfied with the travel accommodation services. However, a past study generated different results, where confirmation significantly influenced tourist satisfaction [35]. But the current study confirmed that tourists who met or exceeded their travel accommodation experiences did not guarantee a feeling of satisfaction.

On the other hand, H12 and H13 were supported and found significant. The effect of confirmation towards price acceptance (H12: β:0.19, p:0.001) reflected tourists’ attitudes in evaluating the worthiness of their overall experience. Moreover, service experience directly influenced tourist satisfaction (H13: β:0.27, p:0.048). The tourists were bound to accept the travel accommodation cost if their uncertainties were reduced and the actual service experience met or exceeded their expectations. In comparison with the study of Lee et al. [13], positive confirmation was significantly associated with price acceptance. Analogous to relevant studies, positive service experience supported travel accommodation’s quality service and competitiveness, resulting in satisfaction [13, 28].

#### 5.1.5 Hypothesis 14 and 15 analysis

Furthermore, H14 was supported because price acceptance directly influenced tourist satisfaction (β:0.70, p:0.001). The price worthiness of accommodations in Palawan was parallel to flexible costs, available bargains, and affordable services and amenities. If the tourists accept the travel accommodation’s cost, they will also feel satisfaction. This result produced a similar outcome to Lee et al. [13] study. Other studies further expounded that price acceptance was significantly and positively associated with continuous visiting behavior [13, 30].

Lastly, H15 was verified because tourist satisfaction directly influenced continuous visiting behavior (β: 0.91, p = 0.001). Relevant studies proved a significant and positive relationship between tourist satisfaction and continuous visiting behavior [13, 30, 38]. This study supported other studies’ claims as well. Tourists who experienced remarkable experiences were more likely to revisit similar travel accommodations in the future. Conversely, dissatisfied tourists have the highest tendency to look for different travel accommodations. Tourists prefer to repeat the same service which they personally experience. They show loyalty if the travel accommodations provide their needs and demands.

### 5.2 Theoretical contribution

Tourists’ feedback affects the operations of travel accommodations. Many tourists feel uncertain when going to a new destination. Hence, it is human nature to seek help from others for validation and approval [54]. The principles of URT govern the aforementioned behavior. URT aims to increase positive experience by predicting future outcomes based on the feedback of experienced individuals [54]. By knowing significant URT constructs, uncertainties of future tourists are lessened. This study also proved that interactive is the most effective method to reduce the uncertainties of tourists. Future tourists are encouraged to apply this method to maximize the positive experience in Palawan’s travel accommodations.

In addition, tourists expect tangible and intangible aspects when traveling to a particular destination. These aspects include travel accommodation amenities, food and beverage, employees’ work ethics, attractions, ancillary services, and physical environment [8, 11, 55, 56]. According to ECT, an individual’s expectation might lead to satisfaction or dissatisfaction [26]. The current study suggested constructs to increase the positive experience of tourists. Since tourists have their preferences, travel accommodations offer numerous services to satisfy them. It has also been identified that Palawan’s travel accommodation constantly strives to aim favorable impressions to the tourists. Therefore, ECT is a proven theory in identifying factors affecting tourists’ continuous visiting behavior.

### 5.3 Practical implication

This study intends to help the stakeholders of Palawan’s tourism industry. The stakeholders identified in this study comprise travel accommodation and their employees, residents, the government, and tourists. Travel accommodation services are further improved through tourists’ feedback. Tourists hold a crucial role because the business demands originate from them. This study determined significant constructs affecting tourists’ continuous visiting behavior. Travel accommodation management and its respective employees can apply significant constructs to satisfy the needs of tourists. If the tourists are satisfied with their travel experiences, they will certainly return to Palawan and avail of services from the same travel accommodation. Travel accommodations must follow the specified guidelines to enhance services that need to be emphasized. They can also mitigate the cost by reducing expenses allocated for insignificant constructs. This implication is a ripple effect because this study also aims to contribute to economic growth. Positive economic growth is equivalent to increased residents’ employment and improved government finances. If there is a continuous tourist demand, business owners will also hire more employees. In return, residents are prioritized in employment opportunities. The government’s revenue is also expected to increase by earning tourists’ loyalty. One scenario could be an additional budget to improve infrastructures within Palawan because the government has political, legislative, and financial power. This study also helps tourists to find the best method when booking travel accommodation. Since it was found that the interactive approach was the most significant method to lessen uncertainties, tourists are encouraged to seek close acquaintances’ feedback to receive truthful responses. Tourists can also mitigate the hours of searching for reviews online from strangers, which might seem unpersuasive. Therefore, these stakeholders are vital in tourism development and tourists’ continuous visiting behavior.

## 6. Conclusion

Palawan tourism has been developing extensive services to satisfy tourists. It has undeniably received high demand from tourism stakeholders. Thus, the need to continuously progress has emerged. Several studies investigated tourist satisfaction, tourist loyalty, and tourist behavior. However, inadequate studies are focusing on travel accommodations situated in Palawan. Hence, this study investigated tourists’ continuous visiting behavior in visiting Palawan’s travel accommodations by applying CB-SEM. URT and ECT governed the CB-SEM technique to further identify the tourists’ behavior.

This study accumulated 712 participants who evaluated Palawan’s travel accommodations. The eleven (11) constructs assessed were passive uncertainty, interactive uncertainty, physical environment, food and beverage, attitude of employees, service experience, perceived performance, confirmation, price acceptance, tourist satisfaction, and continuous visiting behavior. These constructs were connected corresponding to the principles of URT and ECT and produced fifteen (15) hypotheses. Among the identified hypotheses, eleven (11) hypotheses (H2: IU→PP, H3: PE→SE, H5: AE→SE, H6: PE →PA, H8: AE→PA, H9: PP→C, H10: SE→C, H12: C→PA, H13: SE→TS, H14: PA→TS, H15: TS→CVB) were supported by this study. On the other hand, four (4) hypotheses (H1: PU→PP, H4: FB→SE, H7: FB→PA, H11: C→TS) were rejected due to lack of influence. Nonetheless, this study proved that interactive uncertainty greatly influenced perceived performance (H2: IU→PP) compared to passive uncertainty (H1: PU→PP). Physical environment (H3: PE→SE) and attitude of employees (H5: AE→SE) significantly influenced service experience compared to food and beverage (H4: FB→SE and H7: FB→PA). Meanwhile, physical environment produced a negative yet significant impact on service experience (H6: PE →PA). The attitude of employees produced a direct effect on price acceptance (H8: AE→PA). Perceived performance (H9: PP→C) and service experience (H10: SE→C) had a significant and positive effect on confirmation. Moreover, confirmation was insignificant towards tourist satisfaction (H11: C→TS) but was significant and positive towards price acceptance (H12: C→PA). In addition, service experience (H13: SE→TS) and price acceptance positively influenced tourist satisfaction (H14: PA→TS). Lastly, tourist satisfaction strongly influenced continuous visiting behavior (H15: TS→CVB). In coordination with the generated CB-SEM, model fit indices passed the suggested values to validate the strong relationship between the model and data.

This study proved significant constructs bounded by URT and ECT. These two theories helped identify the determinants of tourists’ continuous visiting behavior. It is recommended that tourists follow an interactive approach in choosing a travel accommodation in Palawan to maximize a positive travel experience. The study also emphasized the importance of physical environment and attitude of employees, which indirectly affect tourists’ continuous visiting behavior. Hence, travel accommodations are encouraged to focus on these aspects compared to other amenities and services. Travel accommodations management should allocate a proper budget to enhance the physical environment and properly train the employees. The government is also expected to improve the physical environment by maintaining picturesque sceneries. Future researchers who are also inclined to evaluate Palawan’s travel accommodations can further improve this study. Overall, tourists were expected to continuously visit Palawan’s travel accommodations by maximizing the positive effects of significant constructs. Competition in the tourism sector is inevitable; thus travel accommodations must prioritize improving their services.

The objective of this study was achieved, but limitations were inevitable. Other constructs, measures, and hypotheses were eliminated due to insignificant p-values. Hence, future researchers may include more constructs and measures to strengthen the results of the study. First, COVID-19 safety precaution is highly suggested as an additional construct to discern tourists’ behavior before and during the pandemic. Second, an additional construct related to tourists’ perception of outdoor activities (island hopping, diving, hiking, etc.) could enhance the results. Third, it is suggested to include additional continuous visiting behavior measures; conditional statements should also have refrained. Fourth, it is recommended to pursue collaboration with the Philippines’ Department of Tourism to determine the actual Palawan’s visitor population and demographic profile. Next, an increased number of participants is strongly suggested to diversify demographic characteristics and further improve model fit indices values. Finally, the participants answered the questionnaire through an online platform. Hence, the researchers could not maximize the responses of all ages. Most of the participants’ ages ranged from 18 to 34 years old (75.28%), and the younger generation (≤ 17 years old) and older generation (≥ 35 years old) comprised only 24.72% of the overall participants. These recommendations can immensely help future researchers in producing a more comprehensive study.

## References

[1] Fabinyi M. The role of land tenure in livelihood transitions from fishing to tourism. Maritime Studies. 2020;19(1):29–39. doi: 10.1007/s40152-019-00145-2

[2] Yumol DT. Palawan recognized anew as ’Best Island in the World’: CNN Philippines; 2020 [cited 2021 January 11]. https://www.cnn.ph/lifestyle/2020/7/10/Palawan-recognized-anew-as--best-island-in-the-world-.html.

[3] Padin MG. Palawan targets 2 M tourists in 2017: Philstar Global; 2021 [cited 2021 January 11]. https://www.philstar.com/business/2016/12/07/1650915/palawan-targets-2-m-tourists-2017.

[4] Pe R. Puerto Princesa not about to relax amid tourist influx: Business Mirror; 2017 [cited 2021 January 11]. https://businessmirror.com.ph/2017/12/28/puerto-princesa-not-about-to-relax-amid-tourist-influx/.

[5] Magdayao AG. Visitor arrivals rise 21% in Palawan in 2018: Palawan News; 2019 [cited 2021 January 11]. https://palawan-news.com/visitor-arrivals-rise-21-in-palawan-in-2018/.

[6] Fos PJ. Mimaropa tourist arrivals reach 2.55 million in 2018: Philippine Information Agency; 2019 [cited 2021 January 11]. https://pia.gov.ph/news/articles/1027923.

[7] Cahigas MM, Prasetyo YT, Alexander J, Sutapa PL, Wiratama S, Arvin V, et al. Factors affecting visiting behavior to Bali during the covid-19 pandemic: An extended theory of planned behavior approach. Sustainability. 2022;14(16):10424. doi: 10.3390/su141610424

[8] Bayih BE, Singh A. Modeling domestic tourism: Motivations, satisfaction and tourist behavioral intentions. Heliyon. 2020;6:9. doi: 10.1016/j.heliyon.2020.e04839 32995592 PMC7505762

[9] Philippine Embassy in Paris F. Palawan: parispe.dfa.gov.ph; [cited 2021 May 28]. https://parispe.dfa.gov.ph/consularservices/2015-10-07-09-53-27/86-visit-phl/150-palawan.

[10] Ding K, Choo WC, Ng KY, Ng SI. Employing structural topic modelling to explore perceived service quality attributes in Airbnb accommodation. International Journal of Hospitality Management. 2020;91:102676. doi: 10.1016/j.ijhm.2020.102676

[11] Chen MC, Hsiao YH, Chang KC, Lin MK. Applying big data analytics to support Kansei engineering for hotel service development. Data Technologies and Applications. 2018;53(1):33–57. doi: 10.1108/dta-05-2018-0048

[12] Alonso-Almeida MM, Borrajo-Millan F, Yi L. Are Social Media Data Pushing Overtourism? The Case of Barcelona and Chinese Tourists. Sustainability. 2019;11(12):3356. doi: 10.3390/su11123356

[13] Lee J, Lee H, Chung N. The impact of customers’ prior online experience on future hotel usage behavior. International Journal of Hospitality Management. 2020;91:102669. doi: 10.1016/j.ijhm.2020.102669

[14] Antheunis ML, Valkenburg PM, Peter J. Getting acquainted through social network sites: Testing a model of online uncertainty reduction and social attraction. Computers in Human Behavior. 2010;26(1):100–9. doi: 10.1016/j.chb.2009.07.005

[15] González-Mansilla Ó, Berenguer-Contrí G, Serra-Cantallops A. The impact of value co-creation on hotel brand equity and customer satisfaction. Tourism Management. 2019;75:51–65. doi: 10.1016/j.tourman.2019.04.024

[16] Buhalis D. Marketing the competitive destination of the future. Tourism Management. 2000;21(1):97–116. doi: 10.1016/s0261-5177(99)00095-3

[17] Kusumasondjaja S, Shanka T, Marchegiani C. Credibility of online reviews and initial trust: The roles of reviewer’s identity and review valence. Journal of Vacation Marketing. 2012;18(3):185–95. doi: 10.1177/1356766712449365

[18] Sedera D, Lokuge S, Atapattu M, Gretzel U. Likes—the key to my happiness: The moderating effect of social influence on travel experience. Information & Management. 2017;54(6):825–36. doi: 10.1016/j.im.2017.04.003

[19] Park S, Yang Y, Wang M. Travel distance and hotel service satisfaction: An inverted U-shaped relationship. International Journal of Hospitality Management. 2018;76:261–70. doi: 10.1016/j.ijhm.2018.05.015

[20] Filieri R, Galati F, Raguseo E. The impact of service attributes and category on EWOM helpfulness: An investigation of extremely negative and positive ratings using latent semantic analytics and regression analysis. Computers in Human Behavior. 2021;114:106527. doi: 10.1016/j.chb.2020.106527

[21] Maguigad VM. Tourism planning in Archipelagic Philippines: A case review. Tourism Management Perspectives. 2013;7:25–33. doi: 10.1016/j.tmp.2013.03.003

[22] Roxas B, Chadee D. Effects of formal institutions on the performance of the tourism sector in the Philippines: The mediating role of Entrepreneurial Orientation. Tourism Management. 2013;37:1–12. doi: 10.1016/j.tourman.2012.10.016

[23] Valdez PN, Tupas R, Carol Tan N. “It’s More fun in the Philippines”: Resemiotizing and commodifying the local in tourism discourse. Discourse, Context & Media. 2017;20:132–45. doi: 10.1016/j.dcm.2017.09.002

[24] Berger C, Calabrese R. Some explorations in initial interaction and beyond: Toward a developmental theory of interpersonal communication. Human Communication Research. 1975;1(2):99–112. doi: 10.1111/j.1468-2958.1975.tb00258.x

[25] Ramirez A, Walther JB, Burgoon JK, Sunnafrank M. Information-Seeking Strategies, Uncertainty, and Computer-Mediated Communication. Human Communication Research. 2002;28(2):213–28. doi: 10.1111/j.1468-2958.2002.tb00804.x

[26] Oliver RL. A cognitive model of the antecedents and consequences of Satisfaction Decisions. Journal of Marketing Research. 1980;17(4):460. doi: 10.2307/3150499

[27] Nunkoo R, Teeroovengadum V, Ringle CM, Sunnassee V. Service quality and customer satisfaction: The moderating effects of hotel star rating. International Journal of Hospitality Management. 2019;91:102414. doi: 10.1016/j.ijhm.2019.102414

[28] Al-Refaie A. Effects of human resource management on hotel performance using structural equation modeling. Computers in Human Behavior. 2014;43:293–303. doi: 10.1016/j.chb.2014.11.016

[29] Cahigas MM, Zulvia FE, Ong AK, Prasetyo YT. A comprehensive analysis of clustering public utility bus passenger’s behavior during the COVID-19 pandemic: Utilization of Machine Learning with metaheuristic algorithm. Sustainability. 2023;15(9):7410. doi: 10.3390/su15097410

[30] El-Adly MI. Modelling the relationship between hotel perceived value, customer satisfaction, and customer loyalty. Journal of Retailing and Consumer Services. 2018;50:322–32. doi: 10.1016/j.jretconser.2018.07.007

[31] Roma P, Panniello U, Nigro GL. Sharing economy and incumbents’ pricing strategy: The impact of Airbnb on the hospitality industry. International Journal of Production Economics. 2019;214:17–29. doi: 10.1016/j.ijpe.2019.03.023

[32] Dogru T, Zhang Y, Suess C, Mody M, Bulut U, Sirakaya-Turk E. What caused the rise of Airbnb? An examination of key macroeconomic factors. Tourism Management. 2020;81:104134. doi: 10.1016/j.tourman.2020.104134

[33] Chen H, Bernard S, Rahman I. Greenwashing in hotels: A structural model of trust and behavioral intentions. Journal of Cleaner Production. 2018;206:326–35. doi: 10.1016/j.jclepro.2018.09.168

[34] Pizam A, Milman A. Predicting satisfaction among first time visitors to a destination by using the expectancy disconfirmation theory. International Journal of Hospitality Management. 1993;12(2):197–209. doi: 10.1016/0278-4319(93)90010-7

[35] Xm Fu, Zhang JH, Chan FTS. Determinants of loyalty to public transit: A model integrating satisfaction-loyalty theory and expectation-confirmation theory. Transportation Research Part A: Policy and Practice. 2018;113:476–90. doi: 10.1016/j.tra.2018.05.012

[36] Choi H, Kandampully J. The effect of atmosphere on customer engagement in upscale hotels: An application of S-O-R paradigm. International Journal of Hospitality Management. 2018;77:40–50. doi: 10.1016/j.ijhm.2018.06.012

[37] Pestana MH, Parreira A, Moutinho L. Motivations, emotions and satisfaction: The keys to a tourism destination choice. Journal of Destination Marketing & Management. 2020;16:100332. doi: 10.1016/j.jdmm.2018.12.006

[38] Hung VV, Dey SK, Vaculcikova Z, Anh LT. The influence of tourists’ experience on destination loyalty: A case study of hue city, Vietnam. Sustainability. 2021;13(16):8889. doi: 10.3390/su13168889

[39] Philippine Daily I. In the know: Facebook in ph: INQUIRER.net; 2020 [cited 2020 December 5]. https://newsinfo.inquirer.net/1341781/in-the-know-facebook-in-ph.

[40] Yamane T. Elementary sampling theory: Prentice-Hall Inc; 1967.

[41] Tejada JJ, Punzalan JRB. On the Misuse of Slovin’ s Formula. The Philippine Statistician. 2012;61(1):129–36.

[42] Hair J, Anderson R, Babin B, Black W. Multivariate data analysis: A global perspective: Pearson Upper Saddle River; 2010.

[43] Hair JF, Ringle CM, Sarstedt M. PLS-SEM: Indeed a silver bullet. Journal of Marketing theory and Practice. 2011;19(2):139–52.

[44] Nunnally J, Bernstein I. Psychometric Theory. 3rd ed: McGraw Hill; 1994.

[45] Fornell C, Larcker DF. Evaluating structural equation models with unobservable variables and measurement error. Journal of Marketing Research. 1981;18(1):39. doi: 10.2307/3151312

[46] Hu L, Bentler PM. Cutoff Criteria for Fit Indexes in Covariance Structure Analysis: Conventional Criteria versus New Alternatives. Structural Equation Modeling: A Multidisciplinary Journal. 1999;6:1–55. doi: 10.1080/10705519909540118

[47] Lin CJ, Prasetyo YT, Widyaningrum R. Eye movement parameters for performance evaluation in projection-based stereoscopic display. Journal of Eye Movement Research. 2018;11(6). doi: 10.16910/jemr.11.6.3 33828713 PMC7906757

[48] Gefen D, Straub D, Boudreau M-C. Structural equation modeling and regression: Guidelines for research practice. Communications of the association for information systems. 2000;4(1):7.

[49] Van den Heuvel L, Blicharska M, Stensland S, Rönnbäck P. Been there, done that? effects of centrality-to-lifestyle and experience use history on angling tourists’ loyalty to a Swedish salmon fishery. Journal of Outdoor Recreation and Tourism. 2022;39:100549. doi: 10.1016/j.jort.2022.100549

[50] Marsh HW, Hocevar D. Application of confirmatory factor analysis to the study of self-concept: First- and higher order factor models and their invariance across groups. Psychological Bulletin. 1985;97(3):562–82. doi: 10.1037/0033-2909.97.3.562

[51] Cahigas MM, Prasetyo YT, Persada SF, Nadlifatin R. Examining Filipinos’ intention to revisit Siargao after Super Typhoon Rai 2021 (Odette): An extension of the theory of planned behavior approach. International Journal of Disaster Risk Reduction. 2023;84:103455. doi: 10.1016/j.ijdrr.2022.103455

[52] George D, Mallery M. SPSS for Windows Step by Step: A Simple Guide and Reference 17.0 update. 10a ed: Pearson; 2010.

[53] Philippine Embassy in Aman J. The Filipino People: ammanpe.dfa.gov.ph; [cited 2021 December 10]. https://ammanpe.dfa.gov.ph/2016-06-30-08-29-54/the-filipino-people.

[54] Srull TK, Wyer RS. Person memory and judgment. Psychological Review. 1989;96:58–83. doi: 10.1037/0033-295x.96.1.58 2648446

[55] Sukiman MF, Omar SI, Muhibudin M, Yussof I, Mohamed B. Tourist satisfaction as the key to destination survival in Pahang. Procedia—Social and Behavioral Sciences. 2013;91:78–87. doi: 10.1016/j.sbspro.2013.08.404

[56] Patandianan MV, Shibusawa H. Importance and performance of streetscapes at a tourism destination in Indonesia: The residents’ perspectives. Frontiers of Architectural Research. 2020;9:641–55 doi: 10.1016/j.foar.2020.05.006

